# A set of molecular markers predicts chemosensitivity to Mitomycin-C following cytoreductive surgery and hyperthermic intraperitoneal chemotherapy for colorectal peritoneal metastasis

**DOI:** 10.1038/s41598-019-46819-z

**Published:** 2019-07-22

**Authors:** Nicholas Brian Shannon, Joey Wee-Shan Tan, Hwee Leong Tan, Weining Wang, Yudong Chen, Hui Jun Lim, Qiu Xuan Tan, Josephine Hendrikson, Wai Har Ng, Li Yang Loo, Thakshayeni Skanthakumar, Seettha D. Wasudevan, Oi Lian Kon, Tony Kiat Hon Lim, Grace Hwei Ching Tan, Claramae Shulyn Chia, Khee Chee Soo, Chin-Ann Johnny Ong, Melissa Ching Ching Teo

**Affiliations:** 1 Division of Surgical Oncology, National Cancer Centre Singapore, Singapore, Singapore; 2 Division of Medical Sciences, National Cancer Centre Singapore, Singapore, Singapore; 3 Department of Anatomical Pathology, Singapore General Hospital, Singapore, Singapore

**Keywords:** Colon cancer, Tumour biomarkers

## Abstract

Cytoreductive surgery (CRS) and hyperthermic intraperitoneal chemotherapy (HIPEC) is associated with significant perioperative morbidity and mortality. We aim to generate and validate a biomarker set predicting sensitivity to Mitomycin-C to refine selection of patients with colorectal peritoneal metastasis (CPM) for this treatment. A signature predicting Mitomycin-C sensitivity was generated using data from Genomics of Drug Sensitivity in Cancer and The Cancer Genome Atlas. Validation was performed on CPM patients who underwent CRS-HIPEC (n = 62) using immunohistochemistry (IHC). We determined predictive significance of our set using overall survival as a surrogate endpoint via a logistic regression model. Three potential biomarkers were identified and optimized for IHC. Patients exhibiting lower expression of *PAXIP1* and *SSBP2* had poorer survival than those with higher expression (*p* = 0.045 and 0.140, respectively). No difference was observed in patients with differing *DTYMK* expression (*p* = 0.715). Combining *PAXIP1* and *SSBP2* in a set, patients with two dysregulated protein markers had significantly poorer survival than one or no dysregulated marker (*p* = 0.016). This set independently predicted survival in a Cox regression model (HR 5.097; 95% CI 1.731–15.007; *p* = 0.003). We generated and validated an IHC prognostic set which could potentially identify patients who are likely to benefit from HIPEC using Mitomycin-C.

## Introduction

Colorectal cancer is the third and second most common cancer amongst men and women respectively across the world, accounting for over 690,000 cancer-related deaths on an annual basis^1^. Colorectal peritoneal metastasis (CPM), a condition found synchronously and metachronously in 5% and 19% of patients respectively, was previously regarded as terminal, with a median overall survival (OS) of 12.7 months with palliative systemic chemotherapy^2,3^. Compared to other forms of metastatic colorectal cancer without peritoneal involvement, CPM has consistently demonstrated to have significantly shorter OS despite palliative systemic chemotherapy^4^.

With the advent of cytoreductive surgery (CRS) and hyperthermic intraperitoneal chemotherapy (HIPEC), CPM has seen significant improvements in long-term outcomes, with meta-analyses citing median OS of 34.3 months, average 5-year OS of 40% and a 2-year median recurrence-free survival (RFS)^5,6^. CRS involves a series of peritonectomy procedures and visceral resections that aims to remove all macroscopic disease, following which a heated chemotherapy perfusate is instilled to target remaining viable microscopic disease^7,8^. The use of CRS-HIPEC in the treatment of CPM is currently limited by significant perioperative morbidity rates of 19.8–33.0% and perioperative mortality rates of 2.8–4.3%^6^. It is hence of paramount importance that patients with CPM are carefully selected to undergo CRS-HIPEC for treatment^9^.

In a bid to improve patient selection for CRS-HIPEC, several cohort studies have examined prognostic factors for improved post-operative outcomes and found that clinicopathologic factors such as the Eastern Cooperative Oncology Group (ECOG) score, peritoneal carcinomatosis index (PCI), completeness of cytoreduction (CC) score, lymph node involvement, synchronous liver metastases, tumour differentiation and signet ring cell histology are significant prognostic factors^10–14^. Most of these prognostic factors have limited clinical utility pre-operatively as they can only be determined intra- or post-operatively, which will affect patient selection or counselling.

Therefore, the identification of pre-operative predictive factors will potentially lead to better patient selection and outcomes for CRS-HIPEC. This has been illustrated through the use of targeted therapy, where Trastuzumab is used in breast and gastric malignancies with *Her2/Neu* genomic amplification and overexpression, resulting in improvements in prognosis and patient selection^15,16^. With Mitomycin-C as the pioneer chemotherapeutic agent since the inception of CRS-HIPEC still in widespread clinical use today, the ability to predict tumour chemosensitivity to Mitomycin-C amongst patients with CPM holds the potential to improve patient selection and long-term outcomes for CRS-HIPEC as a treatment modality^6,17–19^.

As such, our study aims to identify pre-operative predictive molecular markers that can be easily assessed in tumour biopsy samples as a surrogate for chemosensitivity to Mitomycin-C in CPM before HIPEC is performed. The conception of a pre-operative set would result in significant influence on patient management.

## Results

### Identification of potential predictive molecular markers

Following a thorough interrogation of the Genomics of Drug Sensitivity in Cancer (GDSC) database, 51 colorectal cancer cell lines were identified. Of these, 38 had Mitomycin-C sensitivity data and 35 were either sensitive or resistant to Mitomycin-C (excluding 3 intermediate sensitivity lines) (Supplementary Table S1). After correlation analysis between the Mitomycin-C IC_50_ and degree of expression of dysregulated genetic markers across these cell lines, we selected the top 10 genes for further analysis (*OR9G1, NLRX1, DTYMK, HMGB1, KCTD15, NF2, PAXIP1, PSG4, SSBP2, ECHDC3*), all with *p* < 0.002.

**Figure 1:**
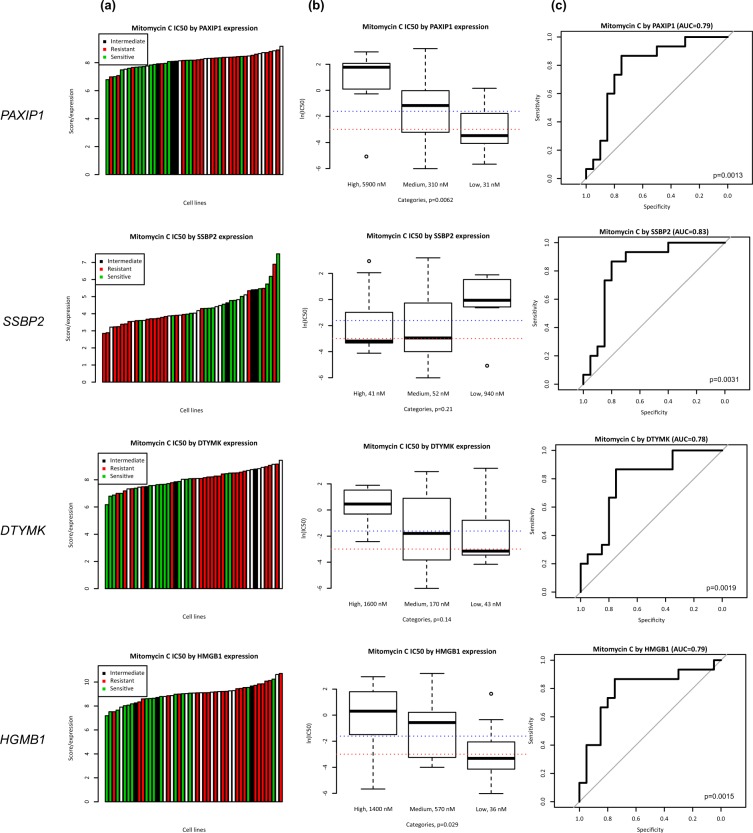
Correlation between gene expression and Mitomycin-C IC_50_ in identified molecular markers. **(a)** Waterfall plot representing distribution of Mitomycin-C sensitivity (resistant IC_50_ > 200 nM, intermediate IC_50_ > 50 nM, and sensitive IC_50_ < 50 nM) across colorectal cancer cell lines ordered by the expression of the respective genes (*PAXIP1, SSBP2, DTYMK, HGMB1*). **(b)** Box plot representing IC_50_ values in cell lines grouped by expression quartiles (high = top quartile, low = bottom quartile, medium = within interquartile range). Dotted lines represent sensitive and resistant cutoffs for IC_50_ values. **(c)** Receiver operating characteristic (ROC) curve representing ability of gene expression to correctly classify sensitive or resistant cell lines (*PAXIP1* AUC = 0.79, *p* = 0.0013, *SSBP2* AUC = 0.83, *p* = 0.0031, *DTYMK* AUC = 0.78, *p* = 0.0019, and *HMGB1* AUC = 0.79, *p = 0.0015*).

**Figure 2:**
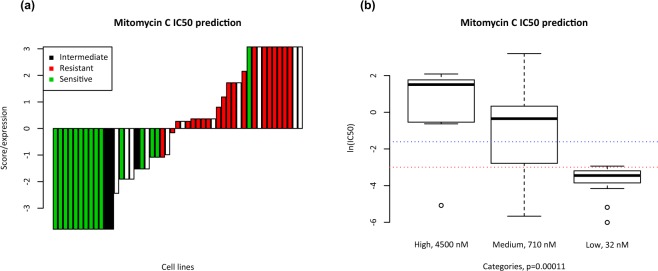
Combined 4-gene predictive model. **(a)** Waterfall plot representing distribution of Mitomycin-C sensitivity (resistant IC_50_ > 200 nM, intermediate IC_50_ > 50 nM, and sensitive IC_50_ < 50 nM) across colorectal cancer cell lines ordered by prediction scores from the combined model. **(b)** Box plot representing IC_50_ values in cell lines grouped by prediction from the 4-gene prediction model (predictions low = sensitive, high = resistant, medium = intermediate score). Dotted lines represent sensitive and resistant cutoffs for IC_50_ values.

Comparison of gene expression with survival (either OS or RFS) across five The Cancer Genome Atlas (TCGA) cohorts (BLCA, COADREAD, CESC, HNSC, OV) allowed us to narrow down the list of genes to four which were significantly associated with RFS and OS (*DTMK, PAXIP1, SSBP2, HMGB1*) in patients treated with cross-linking agents as first-line chemotherapy (Supplementary Table S2). All four of these genes demonstrated correlation between gene expression and Mitomycin-C half maximal inhibitory concentration (IC_50_), and could potentially be used to predict for chemosensitivity to Mitomycin-C (Fig. 1). A combined 4-gene molecular marker set yielded an improved prediction model highly selective for resistant versus sensitive cell lines (*p* < 0.001) (Fig. 2).

### Validation of predictive molecular marker set

To validate our predictive molecular marker set derived from the analysis of the GDSC database, we proceeded to examine the association between level of protein expression (as determined by IHC staining of patient tumour tissue) and OS and disease-free survival (DFS) in our cohort of patients with CPM (n = 62). 3 of the targets (*PAXIP1, SSBP2 and DTYMK*) were successfully optimized for IHC, while 1 target (*HMGB1*) was not successful. The baseline clinical characteristics of our cohort are presented in Table 1. Representative IHC staining of the three targets can be found in Supplementary Fig. S1.

**Table 1:** Demographic and clinical characteristics of patients with CPM (n = 62).

Characteristics	No.	%
**Gender**		
Male	20	32.3
Female	42	67.7
**Race**		
Chinese	51	82.3
Non-Chinese	11	17.7
**Age at surgery, years**		
Median	51.0	
Range	25.0–75.0	
**ECOG Performance Score**		
0–1	57	91.9
2	5	8.1
**PCI score**		
0–20	50	80.6
21–39	6	9.7
**Pathological T stage (8** ^**th**^ **Edition)**		
T1	1	1.6
T3 – T4	59	95.2
**Pathologic N stage (8** ^**th**^ **Edition)**		
N0–N1	39	62.9
N2–N3	20	32.3
**CC score**		
CC0–CC1	61	98.4
CC2–CC3	1	1.6
**Follow-up time, months**		
Median	15.1	
Range	0.4–140.5	
Alive	37	59.7

Note: Sums of numbers may not add up to total number of patients in cohort due to missing data.

Abbreviations: ECOG, Eastern Cooperative Oncology Group; PCI, peritoneal carcinomatosis index; CC, completeness of cytoreduction.

**Figure 3:**
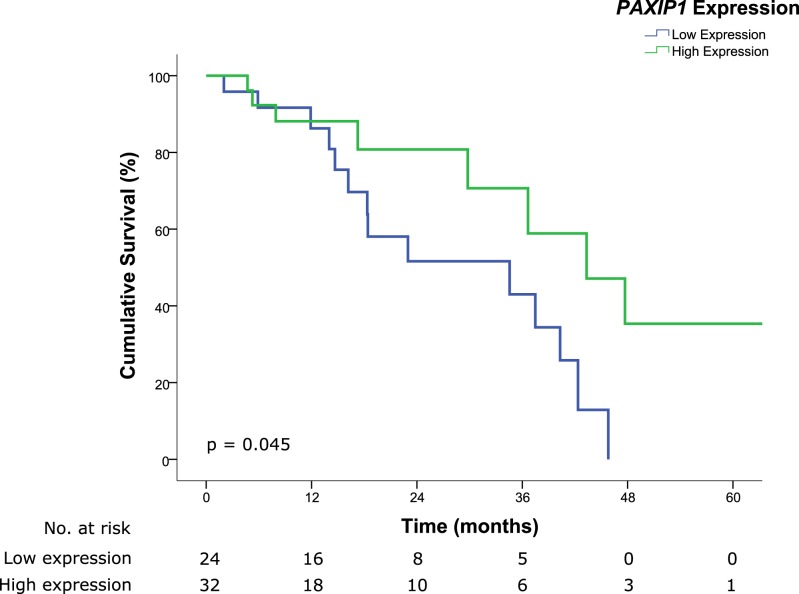
Kaplan-Meier survival curve illustrating poor prognosis in patients with lower expression of *PAXIP1* (median OS, 34.6 months vs. 43.3 months for low and high expressions respectively, HR 2.595, 95% CI 1.022–6.589, *p* = 0.045). Low and high immunoreactivity scores were 0–3 and 4–9, respectively.

**Figure 4:**
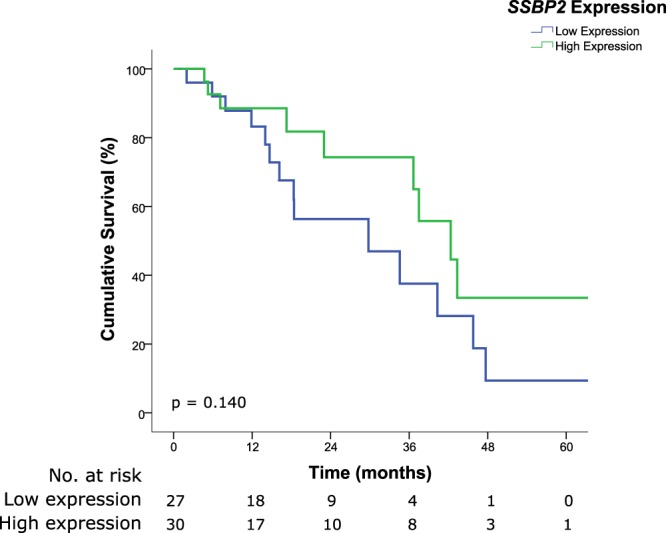
Kaplan-Meier survival curve illustrating poor prognosis in patients with lower expression of *SSBP2* although it did not reach statistical significance (median OS, 29.8 months vs. 42.3 months for low and high expressions respectively, HR 1.886, 95% CI 0.812–4.378, *p* = 0.140). Low and high immunoreactivity scores were 0–3 and 4–9, respectively.

**Figure 5:**
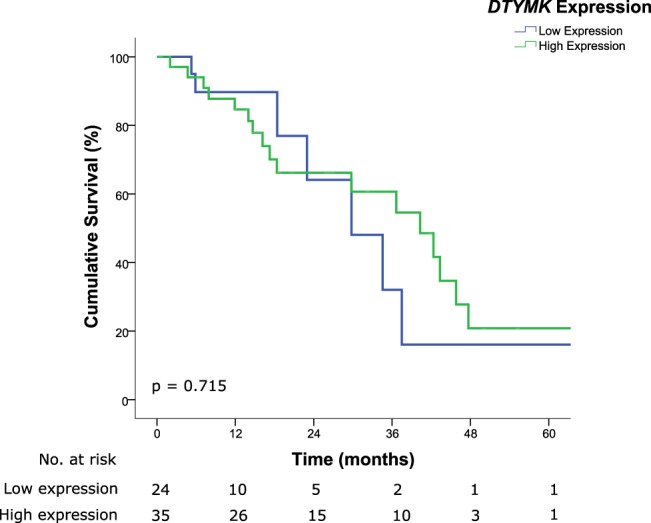
Kaplan-Meier survival curve illustrating no difference in survival in patients with differing expression of *DTYMK* (median OS, 29.8 months vs. 40.3 months for low and high expressions respectively, HR 0.846, 95% CI 0.345–2.077, *p* = 0.715). Low and high immunoreactivity scores were 0–3 and 4–9, respectively.

Survival analysis for each of the markers was performed. Patients exhibiting lower expression of *PAXIP1* had significantly poorer OS as compared to those with higher protein expression (median OS, 34.6 months vs. 43.3 months for low and high expressions respectively, HR 2.595, 95% CI 1.022–6.589, *p* = 0.045) (Fig. 3). Although patients with lower expression of *SSBP2* potentially have poorer OS, this did not reach statistical significance (median OS, 29.8 months vs. 42.3 months for low and high expressions respectively, HR 1.886, 95% CI 0.812–4.378, *p* = 0.140) (Fig. 4). However, no difference was observed in patients for differing levels of expression for *DTYMK* (median OS, 29.8 months vs. 40.3 months for low and high expressions respectively, HR 0.846, 95% CI 0.345–2.077, *p* = 0.715) (Fig. 5). Similar to the OS results, patients with lower expressions of *PAXIP1* (median DFS, 13 months vs. 22 months for low and high expressions respectively, HR 1.935, 95% CI 0.809–4.630, *p* = 0.138) or *SSBP2* (median DFS, 14 months vs. 30 months for low and high expressions respectively, HR 1.913, 95% CI 0.825–4.437, *p* = 0.131) potentially had a shorter DFS as compared to those with higher expressions although it was not statistically significant, while no difference was observed for *DTYMK* (median DFS 14 months vs. 14 months for low and high expressions respectively, HR 1.092, 95% CI 0.441–2.702, *p* = 0.850) (Supplementary Fig. S2).

**Figure 6:**
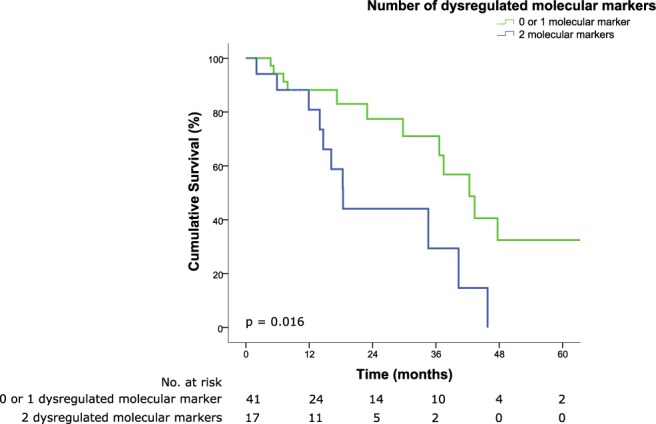
Kaplan-Meier survival curve illustrating poor prognosis in patients with 2 dysregulated molecular markers (median OS, 42.3 months vs. 18.4 months for one or no dysregulated marker and two dysregulated markers respectively, HR 2.844, 95% CI 1.212–6.674, *p* = 0.016).

We next sought to determine the effect of a combined set of *PAXIP1* and *SSBP2* on the prediction for Mitomycin-C chemosensitivity using OS as a surrogate marker. Median OS for patients exhibiting zero, one and two dysregulated protein markers are 43.3, 42.3 and 18.4 months, respectively (*p* = 0.033). The addition of a dysregulated marker results in an increased HR by 1.866 (95% CI 1.052–3.310). Patients were then grouped according to either one or no dysregulated marker versus two dysregulated markers. We found a significant association between the latter and poorer OS, where median survival time was 42.3 months versus 18.4 months (HR 2.844, 95% CI 1.212–6.674, *p* = 0.016) (Fig. 6). A similar association was observed for DFS (median DFS 22 months vs. 12 months for one or no dysregulated marker and two dysregulated markers respectively, HR 2.775, 95% CI 1.191–6.464, *p* = 0.018) (Supplementary Fig. S2).

To confirm that our predictive molecular set was independent of clinicopathologic factors, we performed multivariate Cox regression analysis (Table 2). After adjusting for age, gender, race, ECOG status, PCI score, and pathological T and N stages (8^th^ Edition), the presence of two dysregulated protein markers was found to be independently associated with a poorer prognosis within our cohort (HR 5.097, 95% CI 1.731–15.007, *p* = 0.003).

**Table 2:** Univariate and multivariate survival analyses using the Cox proportional hazard model.

Characteristics	Univariate			Multivariate		
	HR	95% CI	*p*	HR	95% CI	*p*
Age	0.946	0.899–0.995	**0.031**	0.988	0.913–1.069	0.760
**Gender**						
Male	*Ref*			*Ref*		
Female	0.969	0.427–2.200	0.940	1.084	0.354–3.321	0.887
**Race**						
Chinese	*Ref*			*Ref*		
Non-Chinese	3.641	0.854–15.528	0.081	3.285	0.310–34.771	0.323
**ECOG Performance Score**						
0–1	*Ref*			*Ref*		
2	0.673	0.158–2.877	0.594	0.977	0.201–4.747	0.977
**PCI score**						
0–20	*Ref*			*Ref*		
21–39	1.918	0.639–5.753	0.245	2.441	0.578–10.311	0.225
**Pathological T stage (8** ^**th**^ **Edition)**						
T1	*Ref*			*Ref*		
T3 – T4	0.751	0.099–5.704	0.782	0.145	0.013–1.568	0.112
**Pathologic N stage (8** ^**th**^ **Edition)**						
N0–N1	*Ref*			*Ref*		
N2–N3	1.762	0.783–3.968	0.171	2.609	0.746–9.128	0.133
***PAXPI1*** **expression**						
Low (score 0–3) (n = 24, median OS = 34.6 months)	*Ref*			—	—	—
High (score 4–9) (n = 32, median OS = 43.3 months)	2.595	1.022–6.589	**0.045**	—	—	—
***SSBP2*** **expression**						
Low (score 0–3) (n = 27, median OS = 29.8 months)	*Ref*			—	—	—
High (score 4–9) (n = 30, median OS = 42.3 months)	1.886	0.812–4.378	0.140	—	—	—
***DTYMK*** **expression**						
Low (score 0–3) (n = 24, median OS = 29.8 months)	*Ref*			—	—	—
High (score 4–9) (n = 35, median OS = 40.3 months)	0.846	0.345–2.077	0.715	—	—	—
**IHC set**						
0 or 1 dysregulated markers (n = 41, median OS = 42.3 months)	*Ref*			*Ref*		
2 dysregulated markers (n = 17, median OS = 18.4 months)	2.844	1.212–6.674	**0.016**	5.097	1.731–15.007	**0.003**

Abbreviations: HR, hazard ratio; ECOG, Eastern Cooperative Oncology Group; PCI, peritoneal carcinomatosis index; CC, completeness of cytoreduction.

## Discussion

With CRS-HIPEC gaining widespread acceptance as a potential standard of care for patients with CPM, the next frontier to accomplish is to improve long-term outcomes while minimizing perioperative morbidity, a goal in part achievable by tailoring treatment to the unique clinical characteristics and tumour biology of each patient. In the current paradigm of integrative genomic analysis, genomic data can serve as a powerful tool in the identification of patient subgroups for tailoring therapy and prognosis^20^. In addition, the PRODIGE 7 study suggests that the use of a standardized chemotherapeutic regime may not be beneficial for all patients. Instead, a personalized approach may be more suitable^21^.

The key tenets to an effective CRS-HIPEC procedure involve thorough and adequate cytoreduction to achieve maximal surgical debulking, followed by the instillation of intraperitoneal chemotherapy which achieves a peak peritoneal chemotherapeutic agent concentration up to over 1,000 times that achievable by systemic chemotherapy^22^. Mitomycin-C, a primary chemotherapeutic agent of choice for HIPEC in CPM, is subjected to the perennial issue of chemoresistance, best exemplified by the extensive experience in the use of Mitomycin-C in the treatment of bladder cancer^23,24^. Likewise, the ability to predict chemosensitivity to Mitomycin-C through integrative genomic analysis will serve as a potential patient selection and predictive tool in the use of CRS-HIPEC for patients with CPM. There is currently no practical means of routinely assessing chemosensitivity to Mitomycin-C amongst patients with CPM undergoing CRS-HIPEC in a clinical setting. As such, we adopted OS as the primary endpoint in our study, which serves as a surrogate measure of chemosensitivity to Mitomycin-C used in each patient’s CRS-HIPEC procedure.

To this end, we designed our study to identify a set of molecular markers predictive of chemosensitivity to Mitomycin-C by examining the GDSC database, the largest public resource for information on drug sensitivity in cancer cells and molecular markers of drug response^25^. Through correlating Mitomycin-C IC_50_ with the level of gene expression across a wide spectrum of molecular markers for each colorectal cancer cell line, we identified four potential molecular markers with a significant predictive value for chemosensitivity to Mitomycin-C.

To determine the reproducibility of our findings, we then sought to validate the utility of these prognostic markers against an internal cohort of patients with CPM, with a baseline demographic likely dissimilar to that making up the cell lines within GDSC based on a Western population. Of the initial four targets identified, three were successfully optimized on IHC. Subsequently, we found that two of the three markers were prognostic in our patient cohort, and the combined 2-marker set was significantly associated with OS following multivariate Cox regression analysis, outperforming conventional clinicopathologic prognostic factors such as PCI and CC score^10–14^.

Conceding that OS is influenced by many confounders such as other tumour biology factors and the administration and completion of adjuvant systemic chemotherapy, we assessed the effect of these potential predictive molecular markers on DFS. Unsurprisingly, the survival analysis showed similar results to that of OS, suggesting that the predictive markers identified were indeed predictors of chemosensitivity to Mitomycin-C in patients with CPM.

The two predictive markers identified and validated through this study play key biological roles in cellular proliferation. Neither of these genes has been described as potential predictors of Mitomycin-C response in CPM. *SSBP2* and *PAXIP1* are known to be involved in the DNA damage response and aid in maintaining genome stability^26–30^. Specifically, Shaw *et al*.^31^ identified oligodendroglial tumours that responded to chemotherapy had lower expression of *SSBP2*. Correspondingly, *PAXIP1* is involved in the sensitization of lung cancer cells to the WEE1 inhibitor, AZD 1775, in combination with cisplatin^29^. The mechanism of action of cisplatin lies in its association with DNA leading to the formation of DNA adducts, mainly intra-strand DNA adducts, in turn activating multiple signalling pathways and the apoptosis of cells^32^. This is akin to Mitomycin-C, which exerts its anti-tumour activity as a potent DNA cross-linker, resulting in the inhibition of DNA synthesis^33^. Taken together, the roles of *SSBP2* and *PAXIP1* in DNA damage repair support the hypothesis that dysregulation of these molecular markers could confer differential chemosensitivity profiles.

Additionally, literature has shown that *SSBP2* contributes to pathogenesis of some cancers, such as colorectal cancer^34^, prostate cancer^27^, oesophageal squamous cell carcinoma^26^, acute myelogenous leukaemia^28^ and glioblastoma^35^. Likewise, dysregulation of *PAXIP1* expression has been reported to result in poorer patient outcomes. Similar to our findings, lower expression of *PAXIP1* in breast cancer was associated with poorer prognosis^36^. Additionally, upregulation of *PAXIP1* in hepatic cell carcinoma correlated with poorer clinicopathological characteristics, where it was an independent risk factor for poorer OS and RFS^37^.

The major strength of our study is that these predictive molecular markers can be easily assessed for in preoperative tumour biopsy samples through IHC during histological examination in the routine diagnostic and staging evaluation of patients with CPM. The presence of a pre-operative set allows clinicians to predict how a patient would respond to Mitomycin-C even before entering the operating theatre. This would yield additional predictive information to aid in the clinical decision making process to improve patient selection for a morbid procedure such as CRS-HIPEC, enabling better personalized treatment decisions. Moreover, IHC is a simple and cheap tool to achieve reproducible results even in the clinical setting, potentially resulting in a smooth transition from bench to bedside. With the evolution of automated IHC stainers and standardized staining protocols, the testing of this molecular marker set can be easily carried out worldwide. Furthermore, this study utilizes formalin-fixed paraffin-embedded (FFPE) samples to optimize the marker set. These samples are readily available from pathology repositories, providing us with an easy platform for validation. Moving forward, we aim to validate our molecular marker set in a larger patient cohort.

A limitation of our study is the small sample size. Additionally, IHC scoring has a subjective nature. Nonetheless, the presence of two or more blinded scorers interpreting the results limits the subjectiveness involved. There are also an increasing number of computational programs that serve to analyse IHC staining results, although these are associated with their own set of limitations.

## Conclusion

In conclusion, our study identified and validated a molecular marker set that serves as a prognostic factor in CRS-HIPEC patients with CPM, which may be used as a surrogate marker of chemosensitivity to Mitomycin-C and OS. This set can be used in conjunction with other clinicopathologic prognostic factors to guide the optimal management of patients with CPM. However, due to the small sample size, a subsequent larger confirmatory study is required.

## Methods

All experiments were performed in accordance with relevant guidelines and regulations, as approved by the SingHealth Centralized Institutional Review Board (CIRB 2015/2479/F). Informed consent was obtained from participants in this study.

### Identification of potential predictive molecular markers

To identify clinically relevant predictive molecular markers which may predict chemosensitivity to Mitomycin-C, we interrogated the GDSC, an open access database of >1,000 genetically characterized human cancer cell lines screened with a wide range of anti-cancer therapeutics^25^. Each cell line is characterized by varying extents of dysregulated genes, with each specific anti-cancer therapeutic sensitivity data expressed in half maximal inhibitory concentration (IC_50_). An initial list of genes whose expression was predictive of Mitomycin-C sensitivity was identified. Cell lines were grouped by Mitomycin-C sensitivity into resistant (IC_50_ > 200 nM), intermediate (IC_50_ > 50 nM) and sensitive (IC_50_ < 50 nM), and those with intermediate sensitivity were excluded. Genes with expression that was predictive of chemosensitivity were identified for further analysis.

To allow for the selection of molecular predictive markers for chemosensitivity to Mitomycin-C, we next selected genes of prognostic significance on TCGA, a publicly available database of matched transcriptomic and clinical data, to allow cross relevance and to identify predictive markers that is associated with a known clinical endpoint, i.e. OS. As there were a limited number of patients treated with the cross-linking agent Mitomycin-C across the TCGA cohorts, we identified a panel of genes associated with survival in patients treated with platinum agents (as DNA cross-linkers). The number of genes was narrowed down by assessing for genes with survival association in patients treated with platinum agents across the following 5 TCGA cohorts, bladder urothelial carcinoma (BLCA), colorectal adenocarcinoma (COADREAD), cervical squamous cell carcinoma and endocervical adenocarcinoma (CESC), head and neck squamous cell carcinoma (HNSC), and ovarian serous cystadenocarcinoma (OV).

### Study population

The selected molecular predictive markers were validated on a cohort of 62 patients with CPM who underwent CRS-HIPEC at the National Cancer Centre Singapore between April 2001 and February 2017. All treatment decisions were made through a multidisciplinary tumour board comprising surgical, medical and radiation oncologists, radiologists and pathologists.

The extent of peritoneal tumour dissemination was scored at the time of surgery using the peritoneal cancer index (PCI)^38^. CRS was performed to remove all macroscopically visible tumour deposits, which entailed peritonectomy and *en bloc* organ resections where indicated^7^. The residual tumour burden following CRS was evaluated using the completeness of cytoreduction (CC) score: CC-0, no tumour; CC-1, tumour <2.5 mm; CC-2, tumour 2.5–25 mm; CC-3, tumour >25 mm^38^. HIPEC with Mitomycin-C at 10 mg/m^2^ (maximum dose 70 mg) was administered using a closed technique for 60 minutes while maintaining intraperitoneal temperature at 41–43 °C in accordance with the consensus guidelines from The American Society of Peritoneal Surface Malignancies^39^. All HIPEC procedures performed at our institution are done during the same setting as the preceding CRS. Post-operative morbidity and mortality were graded according to the Clavien-Dindo classification^40^.

### Immunohistochemistry

FFPE specimens were cut into 4μm sections and incubated for 1 hour at 60 °C before they were used for IHC staining. Three out of four predictive molecular targets were successfully optimized for IHC with staining performed on the FFPE sections. All IHC was performed using the Bond System (Leica Microsystems, Ltd, Milton Keynes, UK) according to the manufacturer’s recommendations. Antibody sources and optimum IHC staining conditions are described in Supplementary Table S3.

The staining results were determined semi-quantitatively, based on the staining intensity and percentage of tumour cells stained, on a scale of 0 to 9 by at least two independent researchers (JWST, WW and YC) blinded to the outcome. In cases of discrepancies between the score assigned by the researchers, a third researcher (OCAJ) scored the sections independently to determine the final assigned degree of target expression. Staining intensity was stratified into 4 groups; negative (score 0), weak (score 1), moderate (score 2) and strong (score 3) (Supplementary Fig. S1). Similarly, percentage of tumour cells stained was determined as <10% (score 0), 10–50% (score 1), 50–80% (score 2) and >80% (score 3). Scores for staining intensity and percentage of stained tumour cells were multiplied to obtain the final immunoreactivity score. The immunoreactivity scores for each protein target were binarized to low (0–3) and high (4–9) expression.

### Clinical endpoints and statistical analyses

Survival outcomes were examined via Kaplan-Meier analysis. The Cox proportional hazard model was used to examine our target molecular markers against other clinicopathologic variables as predictive factors for OS. All statistical analyses were performed using SPSS (Version 18.0, SPSS Inc., Chicago, USA). A *p*-value of <0.05 was considered statistically significant.

## Supplementary information

Supplementary information

## Data Availability

All data generated or analysed during this study are included in this published article (and its Supplementary Information Files).
