# Supplementary material for: A set of molecular markers predicts chemosensitivity to Mitomycin-C following cytoreductive surgery and hyperthermic intraperitoneal chemotherapy for colorectal peritoneal metastasis
Source: Sci Rep. 2019 Jul 22;9:10572. doi: 10.1038/s41598-019-46819-z (PMC6646658; doi:10.1038/s41598-019-46819-z)
Supplement: Supplementary file 1 — Supplementary information [file 41598_2019_46819_MOESM1_ESM.docx]

**Supplementary Information**

**A set of molecular markers predicts chemosensitivity to Mitomycin-C following cytoreductive surgery and hyperthermic intraperitoneal chemotherapy for colorectal peritoneal metastasis**

Nicholas Brian Shannon^1^*, Joey Wee-Shan Tan^1^*, Hwee Leong Tan^1^*, Weining Wang^1^, Yudong Chen^1^, Hui Jun Lim^1^, Qiu Xuan Tan^1^, Josephine Hendrikson^1^, Wai Har Ng^1^, Li Yang Loo^1^, Thakshayeni Skanthakumar^1^, Seettha D Wasudevan^1^, Oi Lian Kon^2^, Tony Kiat Hon Lim^3^, Grace Hwei Ching Tan^1^, Claramae Shulyn Chia^1^, Khee Chee Soo^1^, Chin-Ann Johnny Ong^1^**, Melissa Ching Ching Teo^1^**

^1^Division of Surgical Oncology, National Cancer Centre Singapore

^2^Division of Medical Sciences, National Cancer Centre Singapore

^3^Department of Anatomical Pathology, Singapore General Hospital

* Equal contribution

** Corresponding authors


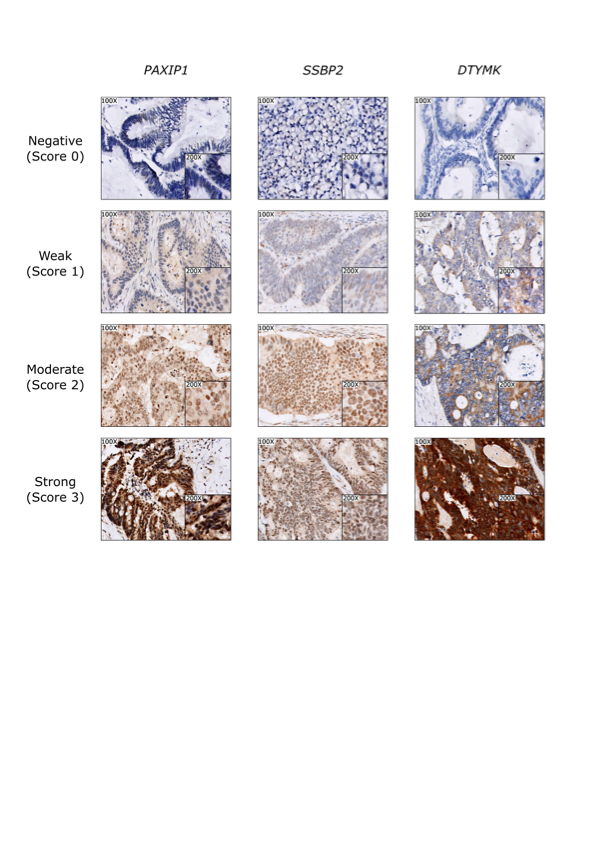


**Supplementary Fig S1.** Representative immunohistochemical (IHC) staining of *PAXIP1*, *SSBP2* and *DTYMK*, illustrating the scores of 0 to 3 for tumour staining intensity scoring in our patient cohort who underwent CRS/HIPEC for CPM.

**Supplementary Fig S2.** Kaplan-Meier disease-free survival (DFS) curves stratified for **(a)** *PAXIP1* (median DFS, 13 months vs. 22 months for low and high expressions respectively, HR 1.935, 95% CI 0.809–4.630, *p*=0.138), **(b)** *SSBP2* (median DFS, 14 months vs. 30 months for low and high expressions respectively, HR 1.913, 95% CI 0.825–4.437, *p*=0.131), **(c)** *DTYMK* (median DFS 14 months vs. 14 months for low and high expressions respectively, HR 1.092, 95% CI 0.441–2.702, *p*=0.850), and **(d)** number of dysregulated genes (median DFS 22 months vs. 12 months for one or no dysregulated marker and two dysregulated markers respectively, HR 2.775, 95% CI 1.191–6.464, *p*=0.018).

| **Supplementary Table S1.** List of colorectal cancer cell lines grouped by sensitivity to Mitomycin-C. | | | | | | | |
| --- | --- | --- | --- | --- | --- | --- | --- |
| **Sensitive** | | **Intermediate** | | **Resistant** | | **Unknown** | |
| **Cell line** | **log IC_50_ (μM)** | **Cell line** | **log IC_50_ (μM)** | **Cell line** | **log IC_50_ (μM)** | **Cell line** | **log IC_50_ (μM)** |
| HCT-116 | -3.47988 | KM12 | -2.41595 | COLO-205 | -0.41062 | NCI-H508 | NA |
| HCT-15 | -3.24404 | COLO-320-HSR | -2.93598 | HT55 | 1.53495 | NCI-H630 | NA |
| HT-29 | -5.18639 | MDST8 | -2.94978 | HT-115 | 1.89632 | SW1116 | NA |
| SW620 | -4.11877 |  |  | LS-513 | -0.62893 | CL-40 | NA |
| HCC2998 | -5.08173 |  |  | NCI-H716 | 2.94946 | CL-34 | NA |
| GP5d | -3.54442 |  |  | RCM-1 | -0.51241 | SNU-1040 | NA |
| LoVo | -4.16299 |  |  | SNU-C2B | 0.45666 | SNU-175 | NA |
| LS-123 | -3.15094 |  |  | SW1417 | 1.64030 | SNU-283 | NA |
| LS-411N | -3.46703 |  |  | SW1463 | -0.56849 | SNU-407 | NA |
| NCI-H747 | -3.19787 |  |  | SW48 | -1.16462 | SNU-61 | NA |
| RKO | -6.01145 |  |  | SW948 | 1.51075 | SNU-81 | NA |
| SK-CO-1 | -3.19005 |  |  | CW-2 | 2.08937 | SNU-C5 | NA |
| SW837 | -3.43011 |  |  | COLO-678 | 2.05786 | DIFI | NA |
| T84 | -4.00056 |  |  | C2BBe1 | -0.27417 |  |  |
| LS-180 | -5.67054 |  |  | SNU-C1 | 3.19947 |  |  |
|  |  |  |  | LS-1034 | 0.15215 |  |  |
|  |  |  |  | CaR-1 | -0.34732 |  |  |
|  |  |  |  | CCK-81 | -0.27214 |  |  |
|  |  |  |  | CL-11 | 2.08099 |  |  |
|  |  |  |  | HCC-56 | 0.21398 |  |  |

| **Supplementary Table S2.** List of genes significantly associated with OS and RFS in patients treated with cross-linking agents as first-time chemotherapy. | | | |
| --- | --- | --- | --- |
| TCGA Cohort | Size of cohort (n) | OS | RFS |
| BLCA | 36 | *DTYMK* | N/A |
| COADREAD | 33 | N/A | *DTYMK, KCTD15* |
| CESC | 129 | N/A | N/A |
| HNSC | 124 | *PAXIP1, SSBP2* | *PAXIP1, SSBP2* |
| OV | 103 | *HMGB1, PAXIP1* | *HMGB1* |
| Abbreviations: OS, overall survival; RFS, recurrence-free survival; BLCA, bladder urothelial carcinoma; COADREAD, colorectal adenocarcinoma; CESC, cervical squamous cell carcinoma and endocervical adenocarcinoma; HNSC, head and neck squamous cell carcinoma; OV, ovarian serous cystadenocarcinoma. | | | |

| **Supplementary Table S3.** Sources of antibodies and immunohistochemical staining conditions for optimum staining. | | | |
| --- | --- | --- | --- |
| **Target** | **Antibody Source** | **Product Number** | **BOND Immunohistochemistry Conditions** |
| *DTYMK* | Abcam, Cambridge, UK | ab154867 | ER1, Bond Refine Kit, Ab 1:200, 20 mins at 25 °C. |
| *SSBP2* | Abcam, Cambridge, UK | ab177944 | ER2, Bond Refine Kit, Ab 1:100, 20 mins at 25 °C. |
| *PAXIP1* | Sigma-Aldrich, Missouri, United States | HPA006694 | ER2, Bond Refine Kit, Ab 1:50, 20 mins at 25 °C. |
